# Role of cytomegalovirus reactivation on relapse after allogeneic hematopoietic stem cell transplantation in acute lymphoblastic leukemia

**DOI:** 10.46989/001c.125912

**Published:** 2024-12-05

**Authors:** Sabrine mekni, Nour Ben Abdeljelil, Rihab Ouerghi, Rimmel yosra kanoun, Siwar Frigui, Dorra Belloumi, Insaf Ben yaiche, Ines Turki, Anna Chabaane, Lamia Torjemane, Tarek Ben Othman

**Affiliations:** 1 Hematology Tunis El Manar University; 2 hematology Tunis El Manar University; 3 hematology Tunis El Manar

**Keywords:** Stem cell transplantation, CMV reactivation, Acute lymphoblastic leukemia, relapse

## Abstract

Cytomegalovirus reactivation (CMV-R) is a frequent complication post-allogeneic hematopoietic stem cell transplantation (allo-HSCT), associated with poor outcomes. Previous studies have demonstrated the protective effect of CMV-R against relapse after allo-HSCT for acute myeloblastic leukemia (AML). However, this impact remains unclear in acute lymphoblastic leukemia (ALL). We conducted a retrospective study on 81 patients with ALL who received allo-HSCT after myeloablative conditioning regimen from matched sibling donors between 2016 and 2022. All patients underwent weekly monitoring for CMV-R by quantitative polymerase chain reaction assay from engraftment until day +100 post allo-HSCT, and received antiviral prophylaxis with acyclovir from day +1 to 6 months after allo-HSCT. Preemptive treatment was initiated when a viremia was higher than 150 copies/mL. The median age was 20 years (range, 5–50 years). After allo-HSCT, 35% of patients developed CMV-R after a median of 39 days (range, 19–100 days). After a median follow-up of 30 months (range, 1-93 months), CMV-R was an independent factor associated with lower cumulative incidence of relapse (CIR) (OR: 0.17; 95% CI [0.03 - 0.98], p = 0.04) without survival benefit. Further studies are needed to validate the protective effect of CMV-R on ALL relapse.

## INTRODUCTION

CMV reactivation (CMV-R) is a common infectious complication after allogeneic hematopoietic stem cell transplantation (allo-HSCT).^1^ Preemptive strategies have been effective in reducing CMV disease, and prophylactic measures by letermovir in CMV seropositive patients resulted in a significantly lower risk of CMV infection.^2,3^ However, CMV-R remains a major cause of morbidity and non-relapse mortality (NRM). In recent studies, CMV-R within 100 days post-allo-HSCT has been associated with a lower relapse incidence in patients with acute leukemia, suggesting a CMV-induced graft-versus-leukemia effect (GVL).^4^

Other studies have shown benefitial effects of CMV-R on relapse in patients transplanted for acute myeloid leukemia (AML).^5,6^ However, its impact on relapse in acute lymphoblastic leukemia (ALL) remains unclear. This study aimed to evaluate the impact of CMV-R on ALL relapse in patients receiving allo-HSCT from HLA-matched sibling donors (MSD).

## METHODS

### Study population

We performed a retrospective study on patients with high-risk ALL who underwent allo-HSCT from a HLA-MSD between January 2016 and December 2022 after a myeloablative conditioning regimen. Patients who had received previous allo-HSCT or had experienced graft failure were excluded.

The study was approved by the local Ethics Committee (CNGMO EC), and all patients and/or legal guardians have signed an informed consent for allo-HSCT.

### CMV monitoring and management

CMV serology was performed in donors and recipients one month before allo-HSCT. All patients received antiviral prophylaxis with acyclovir from day +1 to 6 months after allo-HSCT or longer if they were receiving immunosuppressive therapy. CMV screening was assessed for all patients by real-time quantitative polymerase chain reaction (qPCR) in EDTA-collected plasma (Cobas AmpliPrep/COBAS TaqMan), from engraftment until 100 days after allo-HSCT, with 61 copies/mL as the limit of detection. Preemptive treatment was initiated when qPCR ≥150 copies/mL. Induction therapy consisted of valganciclovir, ganciclovir, or foscarnet based on blood counts, gastrointestinal symptoms, and renal function for at least 14 days, with subsequent adjustments based on viral load response and toxicity, followed by maintenance doses, until two consecutive undetectable qPCR results or in cases of severe toxicity.

### Statistical analysis

The primary endpoint studied was the role of CMV-R on relapse after allo-HSCT in ALL patients. Relapse after allo-HSCT was defined as the presence of ⩾5% of blasts in the bone marrow or cytogenetic, molecular, or clinical relapses. Relapse was calculated as the time from allo-HSCT to relapse, and NRM was calculated as the time from allo-HSCT to death in the absence of prior relapse.

Overall survival (OS) was defined as the time from allo-HSCT to last follow-up or to death from any cause. Relapse-free survival (RFS) was defined as the time from allo-HSCT to relapse or death from any cause.

Chi-square and Fisher’s exact tests were used to compare categorical variables, and the Mann-Whitney or Kruskal-Wallis tests for continuous variables.

The probabilities of OS and RFS were calculated using the Kaplan-Meier method and the log-rank test for univariate comparisons. Acute and chronic GVHD, relapse, and NRM were calculated by using the cumulative incidence (CI). For NRM, relapse was the competing risk, and for relapse, the competing risk was death without relapse. Multivariate analyses for OS and RFS were performed using the Cox proportional hazards regression model and for CIR and CI NRM by the Gray method.

Age, ALL phenotype, cytogenetics, disease status before allo-HSCT, minimal measurable disease (MRD) prior allo-HSCT, EBMT score and HCT-CT score, stem cell source, conditioning regimen, presence of acute GVHD grade ≥ II, chronic GVHD, and CMV reactivation(s) were tested in univariate and multivariate analyses. P-values are two-sided and considered significant when <0.05. Statistical analyses were performed using JAMOVI (version 2.2.5.0).

## RESULTS

### Study population

#### Patient characteristics

A total of 81 patients were included. The median age was significantly higher in patients with CMV-R. Patients and transplant characteristics are summarized in Table 1.

**Table 1.** Patients and transplant characteristics

	All patients (N=81)	CMV-R+ (N=28)	CMV-R- (N=53)	*P*-value
Median age in years (range)	20 (5-50)	31 (5-50)	16 (5-43)	0.012
Age (%)				0.07
<18y	37 (46%)	9 (29%)	28 (53%)	
>=18 y	4 (54%)	19 (71%)	25 (47%)	
Gender (%)				0.87
Male	54 (67%)	19 (68%)	35 (66%)	
Female	27(33%)	9 (32%)	18 (34%)	
Underlying disease (%)				0.65
B-ALL	49 (60%)	16 (57%)	33 (62%)	
T-ALL	32 (40%)	12 (43%)	20 (38%)	
Ph-chromosome (%)				0.12
Ph-chromosome negative	64 (79%)	19 (68%)	45 (85%)	
Ph-chromosome positive	17 (21%)	9 (32%)	8 (15%)	
Cytogenetics (%)				0.11
High-risk	28 (36%)	14 (50%)	14 (28%)	
Standard risk	42 (54%)	11 (40%)	31 (62%)	
Missing	8 (10%)	3 (10%)	5 (10%)	
Disease status (%)				0.75
CR1	68 (84%)	24 (85%)	44 (83%)	
>CR1	13 (16%)	4 (15%)	9 (17%)	
MRD status (%)				0.76
MRD negative (< 10-4)	32 (39.5%)	11 (39%)	21 (39%)	
MRD positive (≥ 10-4)	45 (55.5%)	17 (61%)	28 (53%)	
Missing	4 (5%)	0 (0%)	4 (8%)	
Donor/recipient CMV serostatus (%)				
-/+ or +/+	65 (80%)	22 (78.5%)	43 (81%)	1.0
Others	1 (1.5%)	1 (3.5%)	0 (0%)	
Missing	15 (18.5%)	5 (18%)	10 (19%)	
HCT-CT score (%)				0.33
<2	68(84%)	22 (78.5%)	46 (87%)	
>=2	13 (16%)	6 (21.5%)	7 (13%)	
EBMT score (%)				0.17
<2	46 (57%)	13 (46%)	33 (62%)	
>=2	35 (43%)	15 (54%)	20 (38%)	
Sex mismatch (D/R) (%)				0.50
Female to male	28 (35%)	11(39%)	17 (32%)	
Others	53 (65%)	17 (61%)	36 (86%)	
Median time Diagnosis-allo-HSCT, months (Range)	9 (2-137)	8 (2-80)	10 (3-137)	0.63
Conditioning regimen (%)				0.27
TBI-based	50 (62%)	15 (53%)	35 (67%)	
CT-based	31 (38%)	13 (47%)	18 (33%)	
Stem cell source (%)				0.63
BM	52 (64%)	17 (60%)	35 (67%)	
PB	29 (36%)	11 (40%)	18 (33%)	
GVHD prophylaxis (%)				0.3
Cyclosporine-MTX	58 (72%)	22 (78%)	36 (68%)	
Cyclosporine	23 (28%)	6 (22%)	17 (32%)	
Acute GVHD grade ≥ II (%)				**<10-4**
No	43 (53%)	5 (18%)	38(72%)	
Yes	38 (47%)	23 (82%)	15 (28%)	
Chronic GVHD (%)				0.27
No	50 (62%)	15 (54%)	18 (34%)	
Yes	31 (38%)	13 (46%)	35 (66%)	

ALL:acute lymphoblastic leukemia HR :High-risk ; SR :Standard-risk TBI:Total-body irradiation CR:complete remission ;MRD:measurable residual disease;EBMT:European Bone Transplantation; BM :Bone marrow ;PBSC :peripheral blood stem cells ;GVHD:Graft-versus-host-disease; HCT-CI:hematopoietic comorbidity index ;CMV:Cytomegalovirus; OS:overall survival; RFS -free survival ;NRM: non-relapse mortality

#### CMV reactivation

Among the 81 patients included in the study, 28 (35%) developed CMV-R after a median of 39 days post-allo-HSCT (range, 19–100 days). CMV-R was more frequent in adult patients (>18 years) without being statistically significant (p = 0.07). However, there were no significant differences regarding the other parameters between CMV-R positive and negative patients. CMV-R was significantly higher in patients with acute GVHD grade ≥ II compared to patients without GVHD (82% versus 28%, respectively, p <10^-4^). Among the 28 patients with CMV-R, 20 developed their first CMV-R after the onset of acute GVHD, with a median of 15 days (2-70 days).

#### Relapse, OS, RFS, NRM

**Figure 1.**
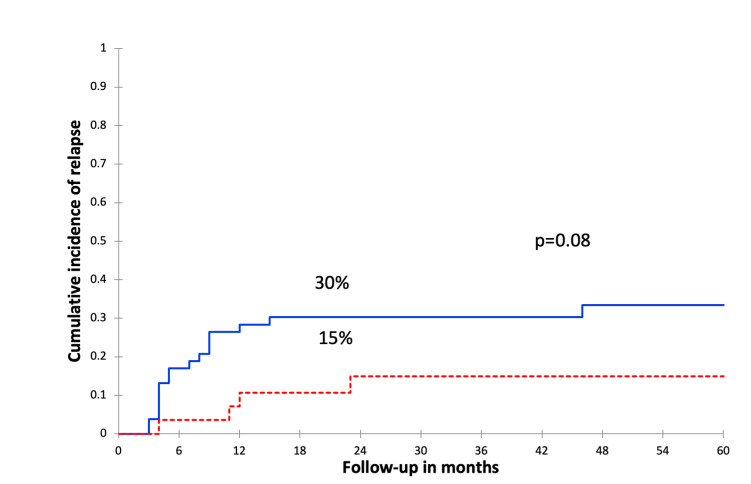
The 2-year cumulative incidence of relapse according to CMV reactivation

At a 30-month median follow-up (range, 1–93 m), 21 (26%) patients relapsed after a median time of 8 months post-allo-HSCT (range, 3–46 m). The 2-year CIR was 25%. Among those patients, 4 relapsed after CMV-R, at a median of 296 days from the first CMV-R (range 79–606d). In univariate analysis, there was a trend to a lower CIR in patients with CMV-R (15% vsersus 30%, p = 0.08, Figure 1). In multivariate analysis, CMV-R was identified as an independent protective factor against relapse (OR = 0.17; 95% CI [0.03 – 0.98], p = 0.04, Table 3).

**Figure 2.**
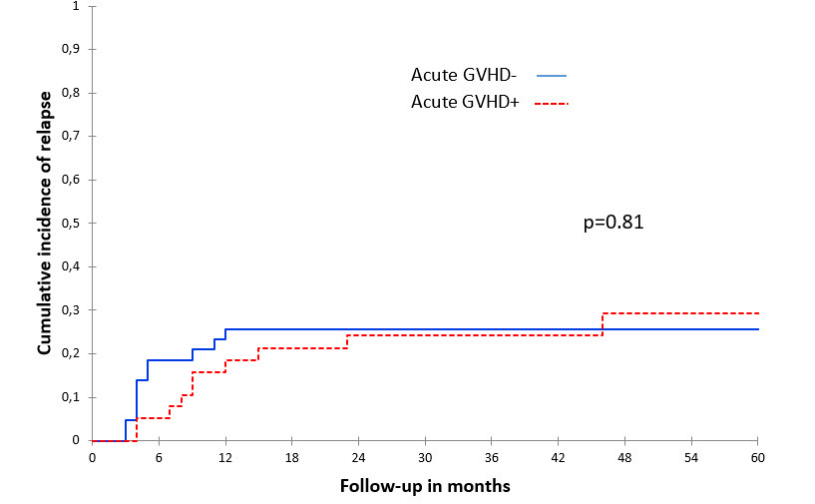
The 2-year cumulative incidence of relapse according to acute GVHD

**Figure 3.**
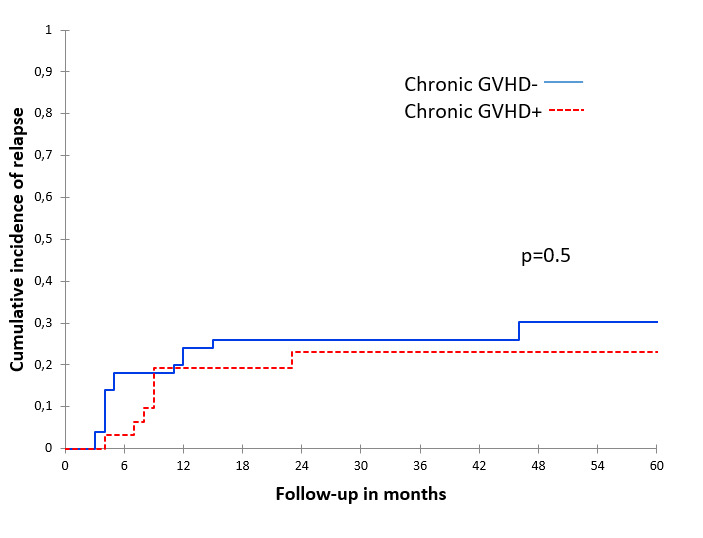
The 2-year cumulative incidence of relapse according to chronic GVHD

There were no significant associations between acute and chronic GVHD and CIR, both in the univariate (Table 2, Figure 2 and Figure 3) and multivariate analyses (Table 3).

**Table 2.** Effect of CMV reactivation on outcomes in univariate analysis

	Relapse		OS		RFS		NRM	
	**%**	**p**	**%**	**p**	**%**	**p**	**%**	**p**
**Age** <18 y >=18 y	24.3% 25.9%	0.76	74.4% 65.1%	0.27	73% 61.3%	0.61	5.4% 15.9%	0.17
**Leukemia phenotype** B-ALL T-ALL	28.6% 21.9%	0.50	70.9% 67.3%	0.8	63.2% 71.9%	0.52	12.2% 9.4%	0.7
**Cytogenetics** SR HR	23.8% 21.4%	0.80	68.3% 80.9%	0.45	66.5% 75%	0.71	9.5% 14.3%	0.70
**Pre- allo-HSCT disease status** CR1 >CR1	26.5% 23.1%	0.8	70.7% 61.5%	0.39	67.6% 61.5%	0.94	8.8% 23.1%	0.10
**MRD** <10-4 >=10-4	25% 26.7%	0.6	73.8% 65.6%	0.41	68.8% 64.4%	0.65	12.5% 11.1%	0.8
**EBMT score** <2 >=2	21.7% 31.4%	0.3	81.8% 53.8%	0.06	76% 54.3%	0.21	**2.2%** **22.9%**	**0.003**
**HCT-CI score** <2 >=2	26.5% 23.1%	0.8	72.4% 53.8%	0.14	69.1% 53.8%	0.99	8.8% 23.1%	0.10
**Conditioning regimen** CT-based TBI-Based	26% 25.8%	0.98	66.4% 71.2%	0.75	70% 61.3%	0.90	10% 12.9%	0.70
**Stem cell source** BM PBSC	25% 27.6%	0.80	71.5% 65.5%	0.21	67.3% 65.5%	0.81	5.8% 20.7%	0.06
**Acute GVHD grade ≥ II** No Yes	25.6% 26.3%	0.81	73.5% 64.4%	0.48	69.8% 62.9%	0.80	**2.3%** **21.1%**	**0.006**
**CMV reactivation(s)** No Yes	30% 15%	0.08	69% 70%	0.77	66% 68%	0.6	**2%** **22%**	**0.02**
**Chronic GVHD** No Yes	17.9% 22.6%	0.50	67.4% 72.5%	0.96	64% 71%	0.5	8% 16.1%	0.25

ALL:acute lymphoblastic leukemia HR :High-risk ; SR :Standard-risk TBI:Total-body irradiation CR:complete remission ;MRD:measurable residual disease;EBMT:European Bone Transplantation; BM :Bone marrow ;PBSC :peripheral blood stem cells ;GVHD:Graft-versus-host-disease; HCT-CI:hematopoietic comorbidity index ;CMV:Cytomegalovirus; OS:overall survival; RFS -free survival ;NRM: non-relapse mortality

**Table 3.** Effect of CMV reactivation on outcomes in multivariate analysis

	Relapse		OS		RFS		NRM		
	**OR** **(95% CI)**	**P**	**OR** **(95% CI)**	**P**	**OR** **(95% CI)**	**P**	**OR** **(95% CI)**	**P**	
**Age** <18 y ≥18 y	1.1 (0.26-4.56)	0.89	1.05 (0.35-3.14)	0.93	1.5 (0.55-4.14)	0.43	0.33 (0.02-4.8)	0.42	
**Cytogenetics** SR HR	0.9 (0.28-4.28)	0.89	0.49 (0.17-1.41)	0.18	0.74 (0.29-1.86)	0.51	1.5 (0.1-21)	0.76	
**Pre-allo-HSCT disease status** CR1 >CR1	0.61 (0.08-4.39)	0.62	1.93 (0.46-8.11)	0.36	1.25 (0.33-4.69)	0.73	0.32 (0.017-6)	0.45	
**EBMT score** <2 ≥2	2.3 (0.63-8.89)	0.19	**4.1** **(1.45 11.54)**	**0.008**	**2.9** **(1.15-7.32)**	**0.02**	14 (0.97-217)	0.05	
**Stem cell source** BM PBSC	1 (0.21-4.31)	0.97	1.52 (0.48-4.79)	0.47	0.93 (0.33-2.67)	0.89	3.43 (0.27-42)	0.33	
**Conditioning regimen** Non TBI based CT-based	1.13 (0.31-4.12)	0.84	0.68 (0.24-1.66)	0.34	0.91 (0.37-2.21)	0.82	3.12 (0.22-43)	0.39	
**Acute GVHD grade ≥ II** No Yes	2.17 (0.5-9.6)	0.30	2.72 (0.9-8.17)	0.08	2.7 (0.96-7.61)	0.06	**11** **(1.26-96)**	**0.03**	
**Chronic GVHD** No Yes	0.87 (0.21-3.56)	0.85	0.7 (0.24-2.3)	0.51	0.60 (0.22-1.62)	0.31	0.51 (0.047-5.57)	0.58	
**CMV reactivation(s)** No Yes	**0.17** **(0.03-0.98)**	**0.04**	0.61 (0.19-1.95)	0.4	0.47 (0.16-1.41)	0.17	0.51 (0.04-5.57)	0.78	

SR:Standard risk, HR:High-risk BM :Bone marrow ;PBSC :peripheral blood stem cells; TBI:Total body irradiation, CT:Chemotheray, GVHD:Graft-versus-host-disease; CMV:Cytomegalovirus; CR:complete remission;EBMT:European Bone Marrow Transplantation;HCT-CI Hematopoietic comorbidity index

**Figure 4.**
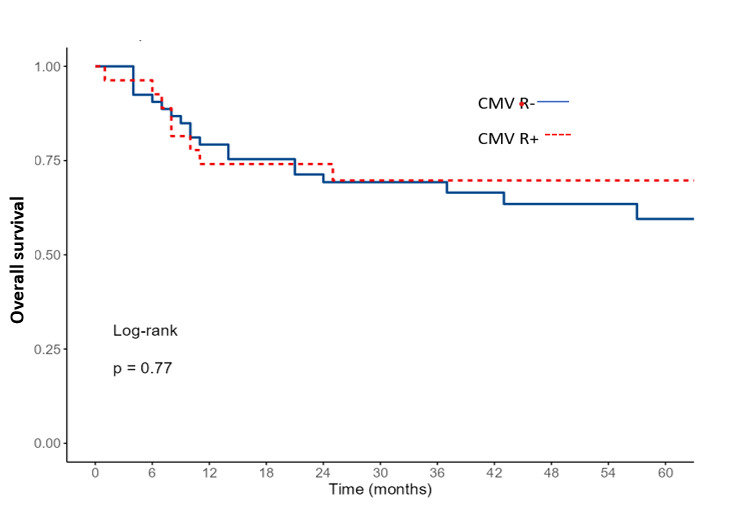
The 2-year overall survival according to CMV reactivation

**Figure 5.**
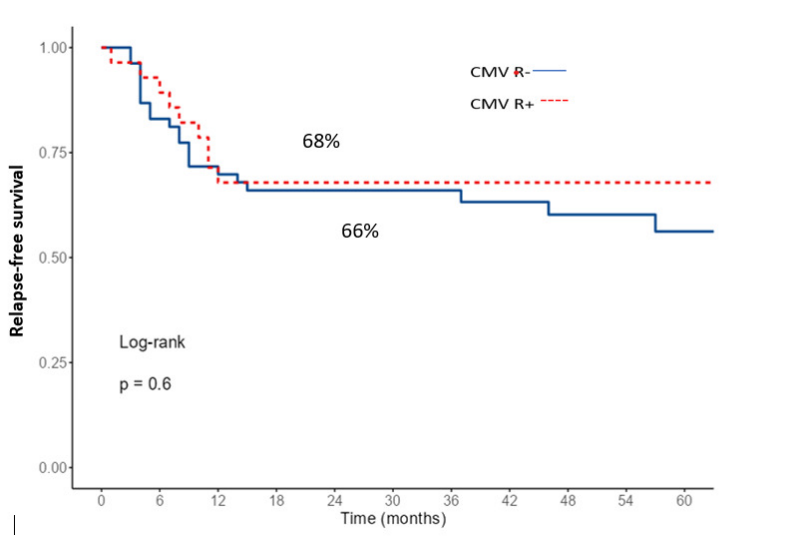
The 2-year relapse-free survival according to CMV reactivation

The 2-year OS and RFS for the entire cohort were 69% and 66%, respectively. When comparing survival according to CMV-R, OS and RFS were similar between the two groups (p=0.77 and p=0.35, respectively, Figures 4 and 5). In multivariate analysis, only an EBMT risk score ≥ 2 was identified as a significant factor for both OS and RFS (Table 3).

**Figure 6.**
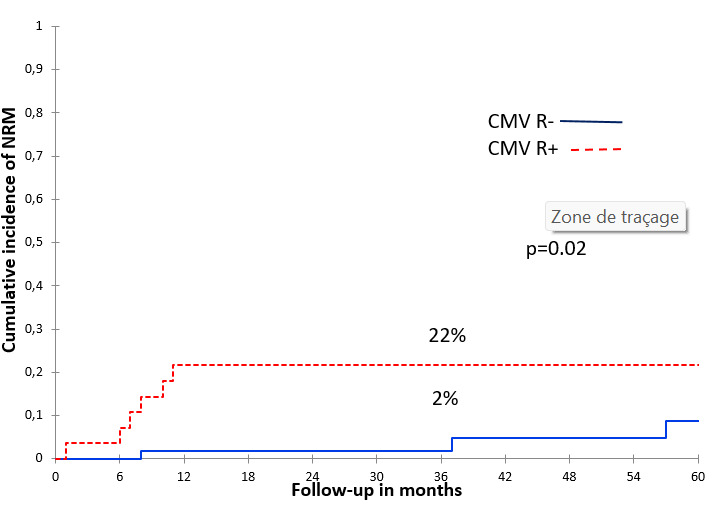
The 2-year cumulative incidence of NRM according to CMV reactivation

CMV-R was significantly associated with increased 2-year NRM only in univariate analysis (Figure 6). No deaths were directly related to CMV-R. However, the NRM was significantly higher in patients with acute GVHD grade ≥ II (OR = 11; IC = 95% [1.26–96], p = 0.03, Table 3). Causes of NRM were pneumonia in 7 patients and sepsis in 2.

## DISCUSSION

CMV-R is a common complication after allo-HSCT and is associated with high morbidity and mortality .^7^ Previous studies have demonstrated the protective effect of CMV-R against relapse after allo-HSCT for patients with AML. However, this impact remains unclear in ALL. The present study aimed to assess this. In our series, CMV-R was significantly associated with reduced CIR. Our findings are in line with previous studies who reported a lower risk of relapse in both AML and ALL in patients developing CMV-R, which is more evident for AML and CML.^5,8,9^ CMV-specific T cells target not only CMV, but also malignant cells infected by CMV, by displaying a CD19-mediated cytolytic function and cytokine production against CD19+ leukemic cells.^10^ These findings suggest that immune cells induced by CMV exposure have the potential to eradicate CMV-infected leukemic cells in both AML and ALL, highlighting the therapeutic potential of CMV-targeted immunotherapy in leukemia treatment.^4^ Furthermore, the natural killer (NK) cells play a pivotal role in controlling CMV-R by targeting infected cells, including malignant cells.^8,9^ Additionally, CMV-R can impact the reconstitution of leukemia-specific T cells and the expansion of specific CD4+ T cell subsets, potentially contributing to the GVL effect and immune protection against pathogens.^8,11–14^

It has been reported that tolerating a low level of CMV viremia and delaying preemptive treatment in those cases may be safe and protective against relapse, supporting the hypothesis that delayed CMV-specific T-cell reconstitution after the use of letermovir could have a negative effect on relapse.^8,15^ In contrast to our results, the study of *Jeljeli et al.* showed that CMV-R was a predictive factor of relapse in children with acute leukemia.^16^ Other reports did not find a significant association between CMV-R and relapse risk.^17–21^ In a large study performed by the Center for International Blood and Marrow Transplant Research (CIBMTR), including 9,469 transplant patients, it was concluded that CMV-R does not confer any protection against hematologic disease relapse.^22^ These findings may be explained by the heterogeneity of the cohort, including different diseases, the use of several methods to detect CMV-R, and the different thresholds for the start of preemptive therapy, which could affect outcomes.

In our study, CMV-R was particularly more frequent in patients with acute GVHD. Hence, the reduced incidence of relapse may be attributed to a more potent GVL effect in patients with acute GVHD, rather than to CMV-R itself. There is a well-established association between GVHD and CMV-R, as the immunodeficiency induced by GVHD and/or its treatment increases the risk of CMV-R.^13,14^ However, it also appears that the causal relationship may, in some cases, be reversed. Indeed, CMV, through its multiple actions on the proliferation of donor alloreactive T lymphocytes, could directly or indirectly trigger the onset of acute GVHD, by exacerbating tissue damage caused by the conditioning regimen, and by increasing the expression of HLA class I and class II molecules^17–21^

There is a well-recognized inverse relationship between GVHD and relapse, as GVHD confers a GVL advantage, which can reduce relapse risk in acute leukemias, highlighting the complex interplay between GVL and GVHD. In our study, acute GVHD was not associated with significantly lower CIR in both univariate and multivariate analyses; however, it was significantly associated with higher NRM, suggesting that the alloimmune balance was tipped toward GVHD. This challenges the traditional association between GVHD and reduced relapse rates. Pilot trials aiming to separate the GVL effect from GVHD after allo-HSCT by isolating and amplifying immune processes that specifically target leukemia cells, thereby preferentially enhancing GVL, are emerging .^22^

In our study, CMV-R had a protective effect against relapse without survival benefit. In fact, the NRM was mostly caused by other infections (bacterial pneumonia, sepsis) occurring after acute GVHD grade ≥ II, and the latter was significantly greater among patients with CMV-R^15,23–26^

Our study has several limitations. First, it is a retrospective cohort, including a limited sample size. Molecular profiles and MRD, which are major predictive factors for relapse in ALL, were not assessed for all patients. The threshold for preemptive treatment was different from other studies. The duration of antiviral treatment and the viral clearance were not specified. There was no monitoring of immune reconstitution post-allo-HSCT, particularly regarding the CMV-specific QuantiFERON assay.

However, the cohort included a homogeneous population (ALL) with a myeloablative conditioning regimen for all patients. Additionally, the PCR test was standardized for a well-defined preemptive treatment.

In conclusion, CMV-R was associated with reduced risk of relapse in patients with ALL without survival benefit, which was explained by the increased incidence of NRM in these patients. Further studies are needed to validate the protective effect of CMV-R on ALL relapse.

### Statement and declarations

The authors declare no competing financial interests
